# Supplementary material for: Multimodal data fusion using sparse canonical correlation analysis and cooperative learning: a COVID-19 cohort study
Source: NPJ Digit Med. 2024 May 7;7:117. doi: 10.1038/s41746-024-01128-2 (PMC11076490; doi:10.1038/s41746-024-01128-2)
Supplement: Supplementary file 1 — Supplementary Tables and Figures [file 41746_2024_1128_MOESM1_ESM.pdf]

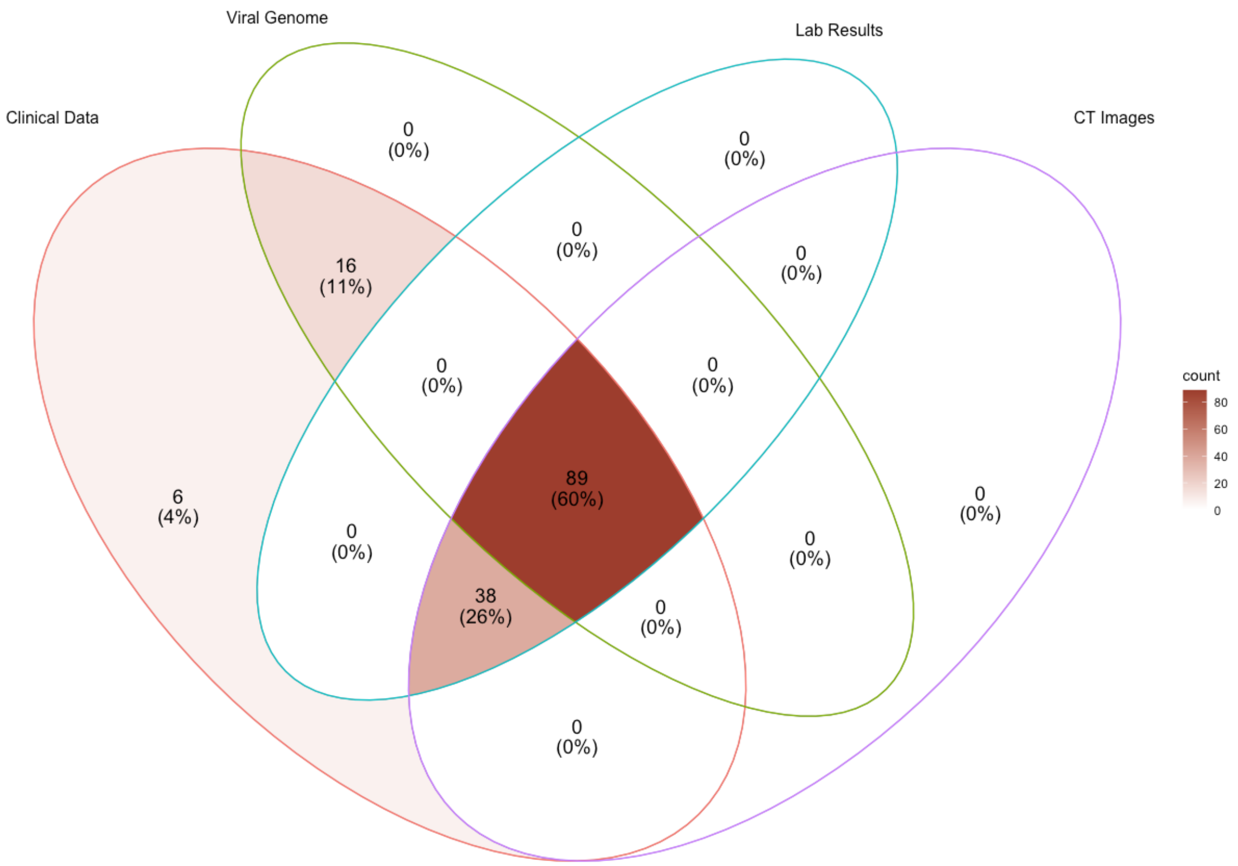

Supplementary Figure 1: The number of patients in the cohort with different data modalities

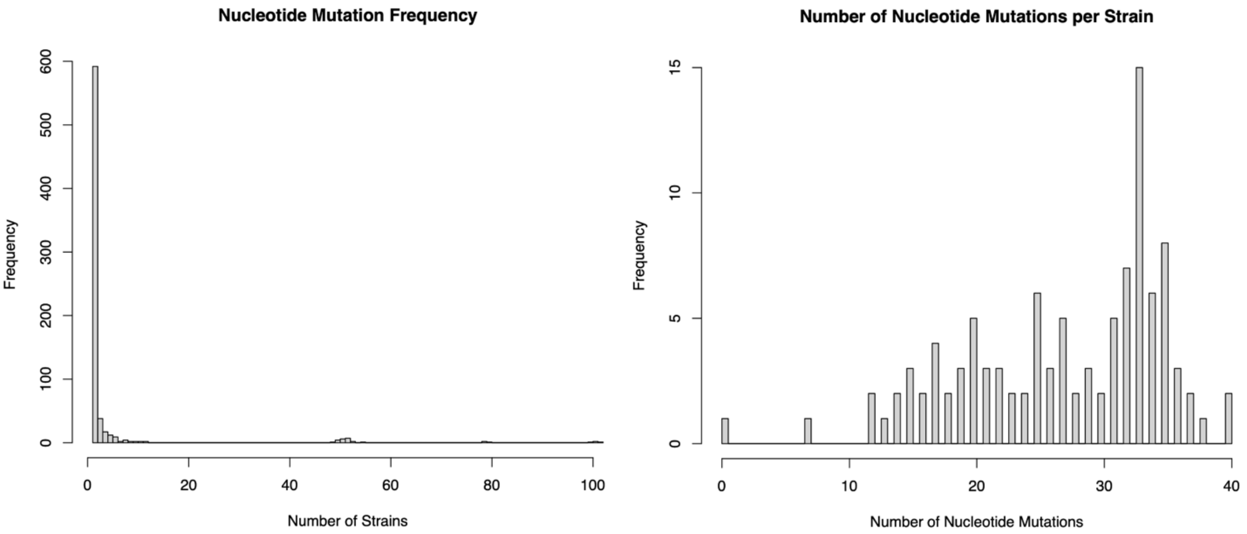

Supplementary Figure 2: Nucleotide mutation frequency and number of nucleotide mutations per strain histograms. The histogram on the left side shows the nucleotide mutation frequency. 592 nucleotide mutations were observed at once. The histogram on the right side shows the number of nucleotide mutations per strain.

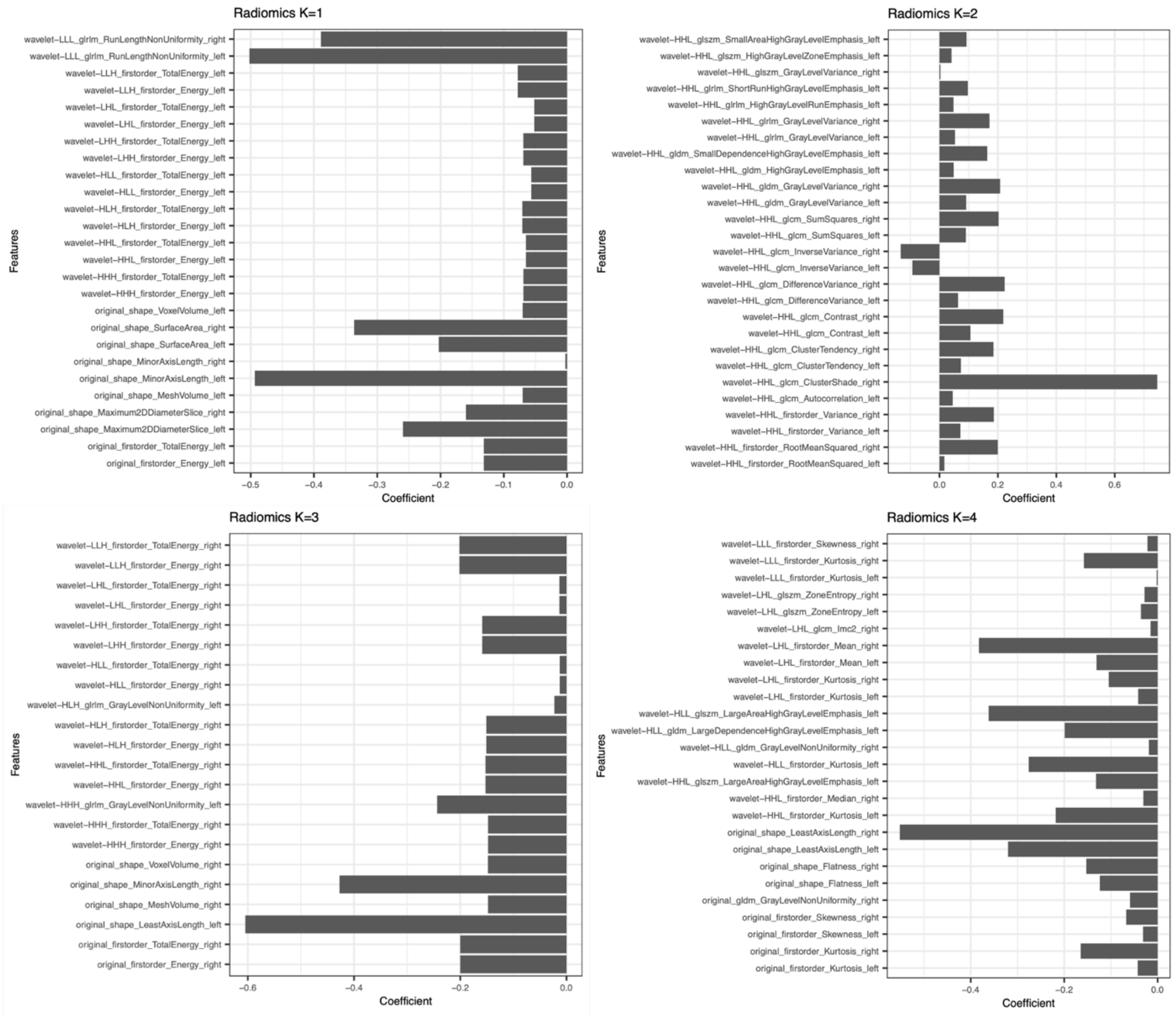

Supplementary Figure 3: Sparce CCA analysis of imaging and clinical data. The first four canonical variates are provided. The first and third canonical vectors are found to be associated with sex, and the second and fourth ones were with lymphoma and CCI, respectively.

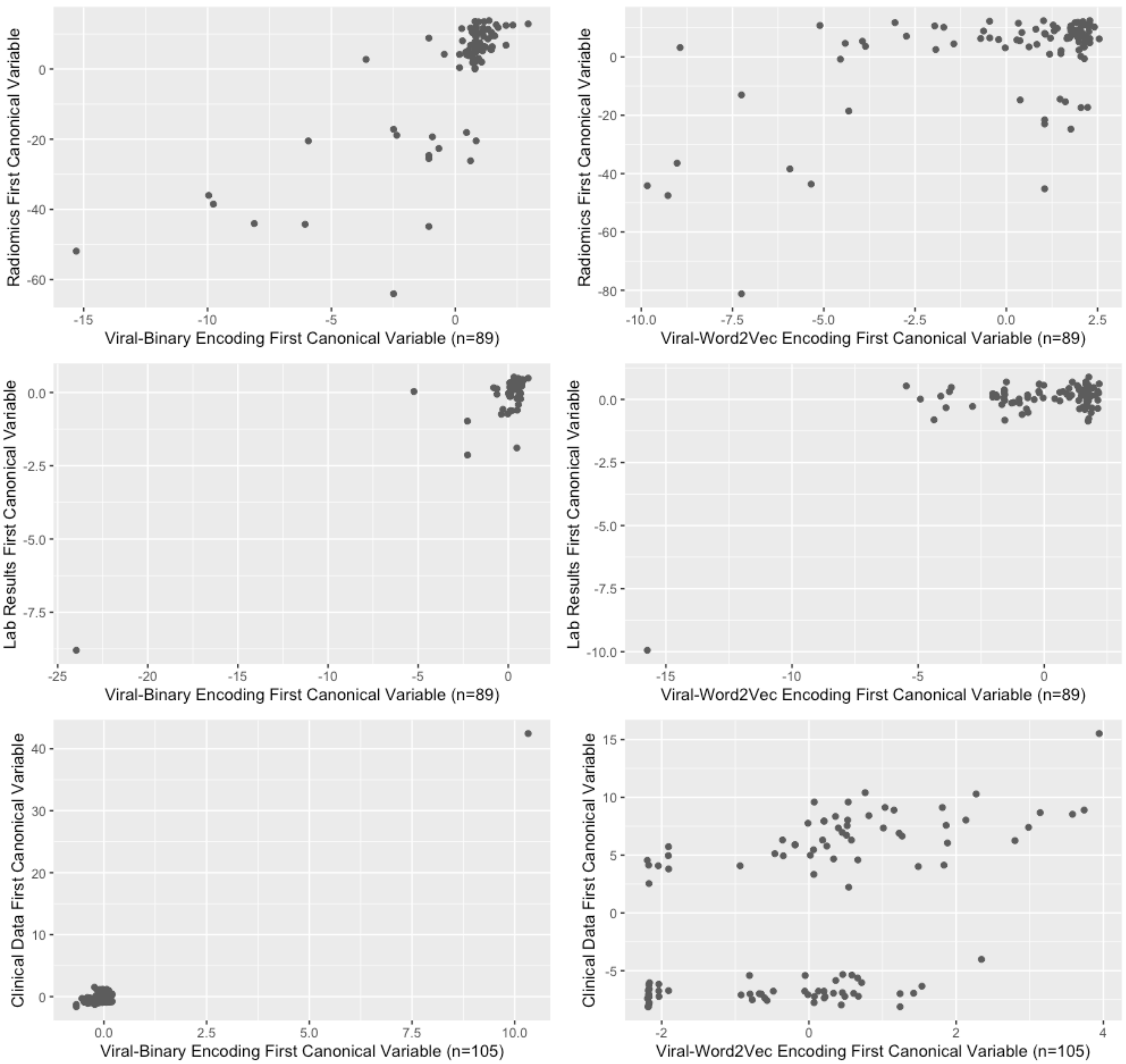

Supplementary Figure 4: Sparce CCA analysis of viral genome sequencing data with different data modalities. The left side shows the first canonical variables of Viral-Binary encoding, and the right side shows Viral-Word2Vec encoding. As it is seen, different encoding techniques for the viral genome change the correlation plots, yielding a better separation with Viral-Word2Vec encoding. Only the correlation plot at the bottom left was found to be significant ($p<0.01$).

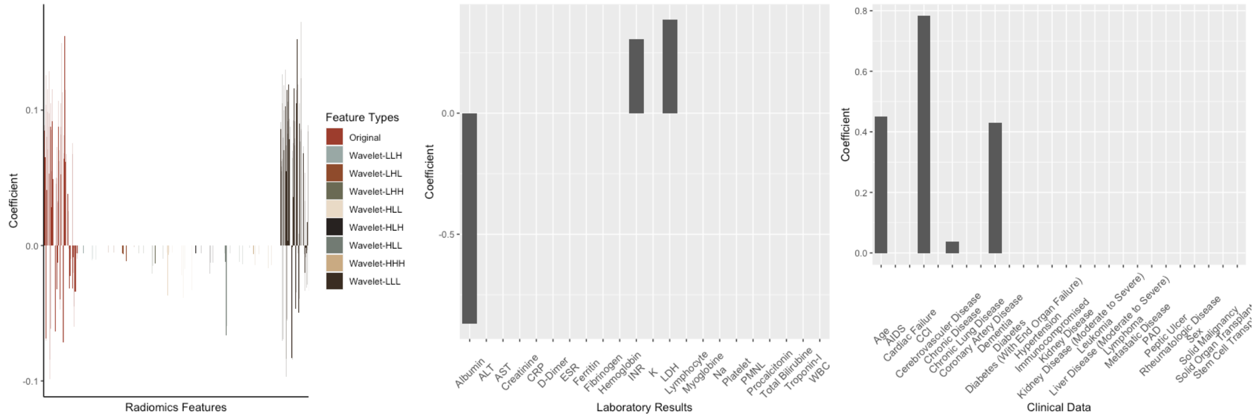

Supplementary Figure 5: The first canonical vectors of multi-CCA. While the dominance of histogram-based first-order radiomics features and the importance of albumin and LDH among the laboratory results persist in the analysis of these 89 patients, as seen in the example of sparse CCA analysis of radiomics features and laboratory results, clinical data's first canonical vectors slightly reveal a different clinical phenotype of an elderly multimorbid patient with dementia.

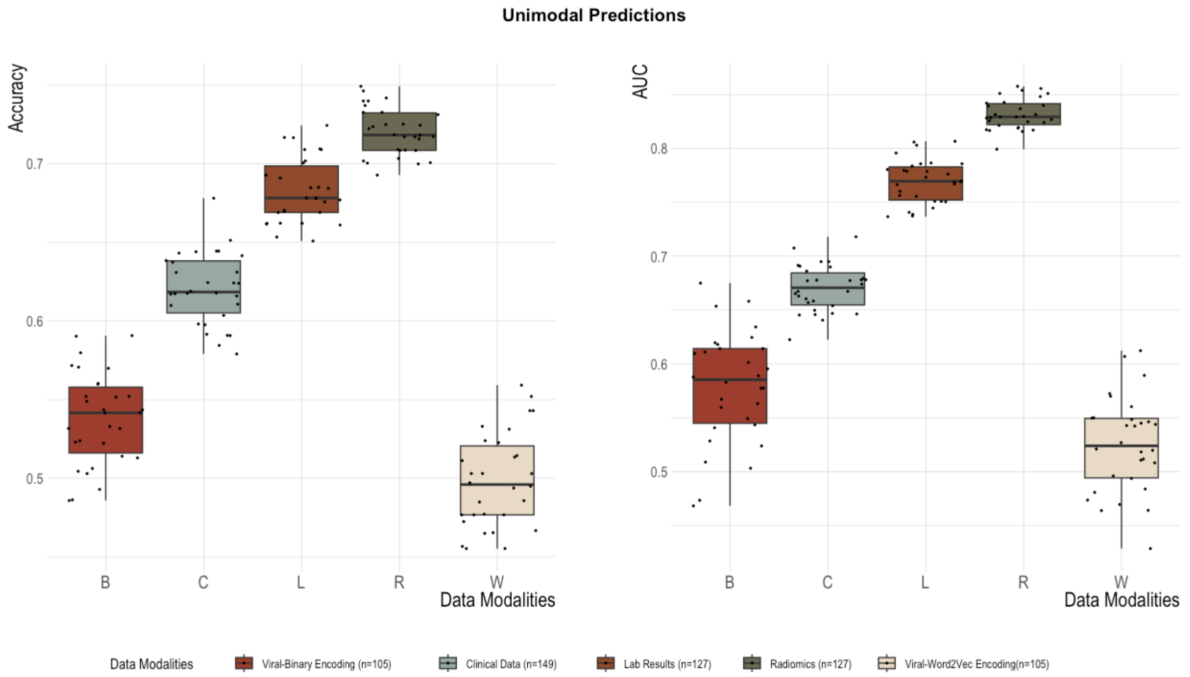

Supplementary Figure 6: Unimodal prediction models for all data modalities in all available patients. Radiomics features have the highest score (AUC=0.83 ± 0.01), followed by laboratory results and clinical data (AUC=0.77 ± 0.02 vs. 0.67 ± 0.02), respectively

**Supplementary Table 1: Unimodal Prediction Models Evaluated in All Available Patients**				
**Model**	***n***		**Accuracy**	**AUC**
	**ICU (+)**	**ICU (-)**		
Clinical Data	63 (42%)	86 (58%)	0.62 ± 0.02	0.67 ± 0.02
Radiomics Features	57 (45%)	70 (55%)	0.72 ± 0.02	0.83 ± 0.01
Laboratory Results	57 (45%)	70 (55%)	0.68 ± 0.02	0.77 ± 0.02
Viral-Binary Encoding	49 (47%)	56 (53%)	0.54 ± 0.03	0.58 ± 0.05
Viral-Word2Vec Encoding	49 (47%)	56 (53%)	0.50 ± 0.03	0.52 ± 0.04
Abbreviations: ICU, Intensive care unit: AUC, Area under the curve				

Supplementary Table 2: Unimodal and Multimodal Prediction Models Evaluated in 89 Patients with Post-hoc Analysis Results (n=89)

Model	Accuracy	Adjusted p-values															
		C	L	R	D	W	CL	CR	LR	CD	CW	LD	LW	RD	RW	CLRD	CLRW
**C**	0.63 ± 0.04	-	.000	.000	.000	.000	.000	.000	.000	.138	.167	.000	.000	.000	.000	.000	.000
**L**	0.74 ± 0.03	.000	-	.874	.000	.000	.366	.03	.958	.000	.000	.85	.824	.999	.774	1	.005
**R**	0.72 ± 0.03	.000	.874	-	.000	.000	.003	.731	.082	.000	.000	1	1	1	.019	.693	.000
**B**	0.55 ± 0.03	.000	.000	.000	-	1	.000	.000	.000	.036	.000	.000	.000	.000	.000	.000	.000
**W**	0.55 ± 0.04	.000	.000	.000	1	-	.000	.000	.000	.148	.003	.000	.000	.000		.000	.000
**CL**	0.76 ± 0.03	.000	.366	.003	.000	.000	-	.000	1	.000	.000	.003	.004	.03	1	.431	.936
**CR**	0.71 ± 0.04	.000	.03	.731	.000	.000	.000	-	.000	.000	.000	.775	.906	.311	.000	.013	.000
**LR**	0.75 ± 0.03	.000	.958	.082	.000	.000	1	.000	-	.000	.000	.074	.08	.361	1	.981	.383
**CB**	0.59 ± 0.05	.138	.000	.000	.036	.148	.000	.000	.000	-	1	.000	.000	.000	.000	.000	.000
**CW**	0.60 ± 0.04	.167	.000	.000	.000	.003	.000	.000	.000	1	-	.000	.000	.000	.000	.000	.000
**LB**	0.72 ± 0.03	.000	.85	1	.000	.000	.003	.775	.074	.000	.000	-	1	1	.017	.658	.000
**LW**	0.72 ± 0.03	.000	.824	1	.000	.000	.004	.906	.08	.000	.000	1	-	1	.021	.642	.000
**RB**	0.73 ± 0.03	.000	.999	1	.000	.000	.03	.311	.361	.000	.000	1	1	-	.132	.987	.000
**RW**	0.75 ± 0.03	.000	.774	.019	.000	.000	1	.000	1	.000	.000	.017	.021	.132	-	.844	.567
**CLRB**	0.74 ± 0.03	.000	1	.693	.000	.000	.431	.013	.981	.000	.000	.658	.642	.987	.844	-	.006
**CLRW**	0.77 ± 0.03	.000	.005	.000	.000	.000	.936	.000	.383	.000	.000	.000	.000	.000	.567	.006	-

Model	AUC	Adjusted p-values															
		C	L	R	D	W	CL	CR	LR	CD	CW	LD	LW	RD	RW	CLRD	CLRW
**C**	0.66 ± 0.03	-	.000	.000	.000	.000	.000	.000	.000	.083	.03	.000	.000	.000	.000	.000	.000
**L**	0.83 ± 0.03	.000	-	1	.000	.000	.849	.134	1	.000	.000	.019	.329	1	.001	1	.000
**R**	0.83 ± 0.03	.000	1	-	.000	.000	.593	.354	.993	.000	.000	.064	.592	1	.002	1	.000
**B**	0.59 ± 0.05	.000	.000	.000	-	.24	.000	.000	.000	.17	.375	.000	.000	.000	.000	.000	.000
**W**	0.55 ± 0.05	.000	.000	.000	.24	-	.000	.000	.000	.000	.000	.000	.000	.000	.000	.000	.000
**CL**	0.85 ± 0.03	.000	.849	.593	.000	.000	-	.000	.015	.000	.000	.000	.006	.862	.972	.665	.462
**CR**	0.81 ± 0.02	.000	.134	.354	.000	.000	.000	-	.999	.000	.000	.999	1	.008	000	.304	.000
**LR**	0.84 ± 0.03	.000	1	.993	.000	.000	.015	.999	-	.000	.000	.002	.069	1	.219	.997	.03
**CB**	0.62 ± 0.04	.083	.000	.000	.17	.000	.000	.000	.000	-	1	.000	.000	.000	.000	.000	.000
**CW**	0.62 ± 0.05	.03	.000	.000	.375	.000	.000	.000	.000	1	-	.000	.000	.000	.000	.000	.000
**LB**	0.80 ± 0.03	.000	.019	.064	.000	.000	.000	.999	.002	.000	.000	-	1	.001	.000	.052	.000
**LW**	0.81 ± 0.03	.000	.329	.592	.000	.000	.006	1	.069	.000	.000	1	-	.085	.000	.539	.000
**RB**	0.83 ± 0.02	.000	1	1	.000	.000	.862	.008	1	.000	.000	.001	.085	-	.000	1	.000
**RW**	0.86 ± 0.02	.000	.001	.002	.000	.000	.972	000	.219	.000	.000	.000	.000	.000	-	.003	.956
**CLRB**	0.83 ± 0.02	.000	1	1	.000	.000	.665	.304	.997	.000	.000	.052	.539	1	.003	-	.000
**CLRW**	0.87 ± 0.03	.000	.000	.000	.000	.000	.462	.000	.03	.000	.000	.000	.000	.000	.956	.000	-

Abbreviations: ICU, Intensive care unit; AUC, Area under the curve; C, Clinical data; L, Laboratory results; R, Radiomics; B, Viral-Binary encoding; W, Viral-Word2Vec encoding

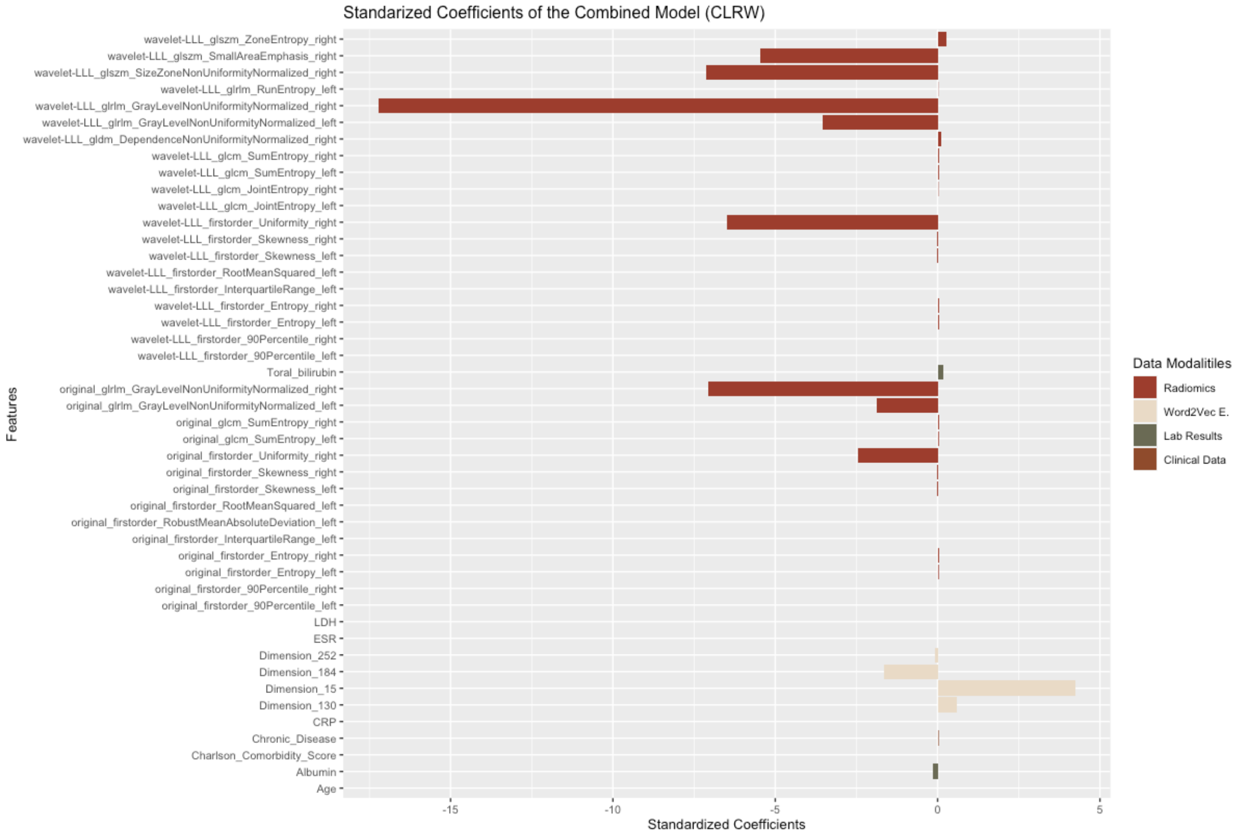

Supplementary Figure 7: Standardized coefficients of the quadruple model (CLRW). Model standardized coefficients were extracted at λ = 0.1. The original and wavelet features in the LLL frequency channel have the highest absolute values for the standardized coefficients. Word2Vec embedding also contributes to the supervised task with its four dimensions.

Supplementary Note 1: Viral Genome Data Availability. All genome sequences and associated metadata in this dataset are published in GISAID's EpiCoV database. To view the contributors of each individual sequence with details such as accession number, Virus name, Collection date, Originating Lab and Submitting Lab and the list of Authors, visit 10.55876/gis8.231104eq. EPI_SET_231104eq is composed of 653,134 individual genome sequences. The collection dates range from 2019-12-30 to 2023-03-02. Data were collected in 171 countries and territories. All sequences in this dataset are compared relative to hCoV-19/Wuhan/WIV04/2019 (WIV04), the official reference sequence employed by GISAID (EPI_ISL_402124). Learn more at https://gisaid.org/WIV04.

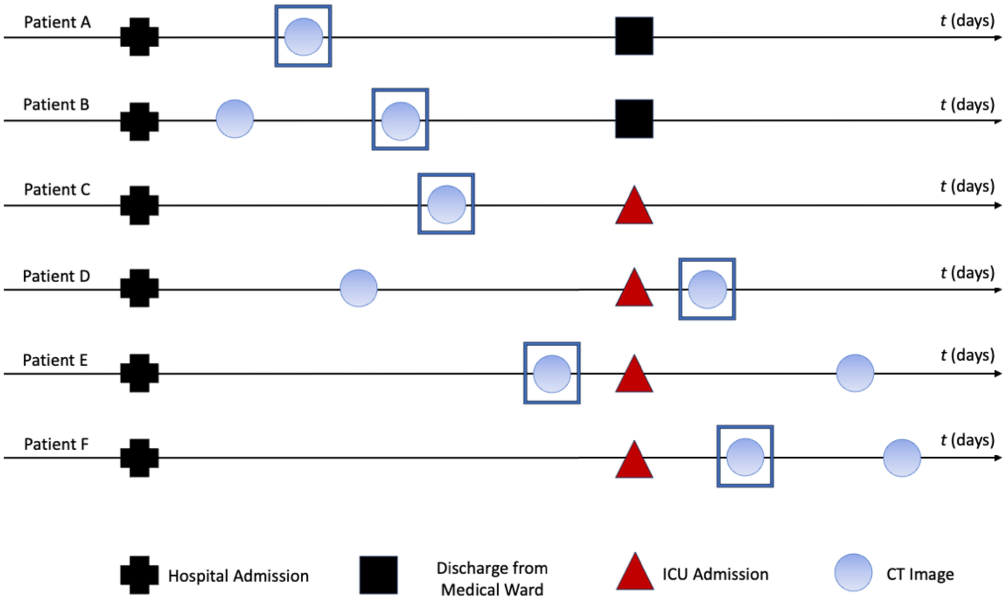

Supplementary Figure 8: CT Image Selection Process. Patients A and B are examples of the non-ICU group, whereas Patients C, D, E, and F are examples of the ICU group. The selected CT images for the study are shown in the frame. If a patient has more than one CT image, images closest to the discharge date from the medical ward or ICU admission were selected. For the sake of simplicity, hospital admission and outcome dates are aligned.

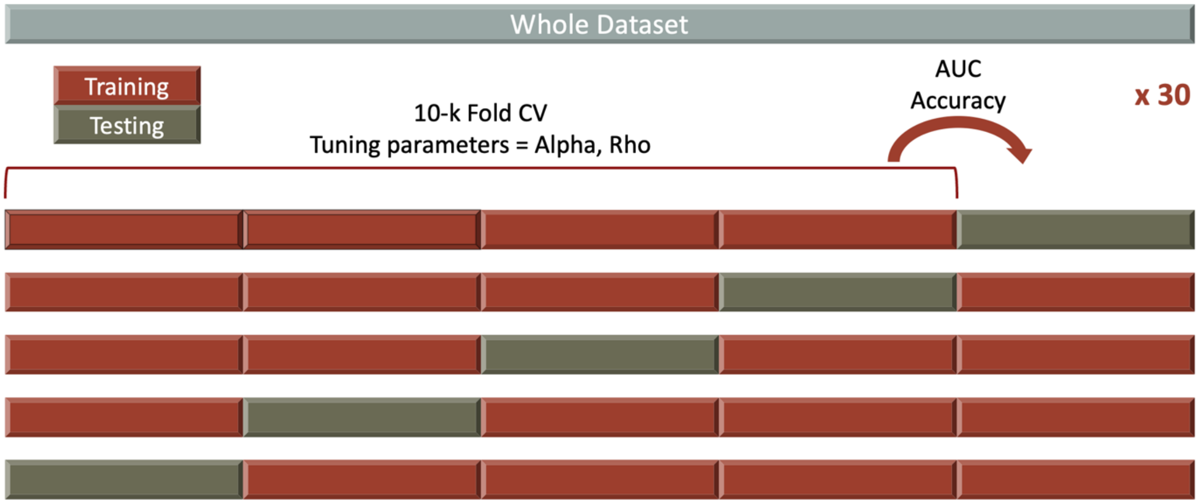

Supplementary Figure 9: Repeated stratified nested cross-validation. 10-fold cross-validation (CV) was performed, with the loss function as "deviance" for tuning the elastic-net mixing parameter and the weight of the agreement penalty in the inner loop. The outer loop assessed the performance of models trained in the inner loop. AUC and accuracy metrics were calculated. The final performance scores were averaged after a 5-fold CV. We conducted each experiment 30 times.
