# Supplementary material for: Multimodal data fusion using sparse canonical correlation analysis and cooperative learning: a COVID-19 cohort study
Source: NPJ Digit Med. 2024 May 7;7:117. doi: 10.1038/s41746-024-01128-2 (PMC11076490; doi:10.1038/s41746-024-01128-2)
Supplement: Supplementary file 2 — Reporting Summary [file 41746_2024_1128_MOESM2_ESM.pdf]

nature portfolio

Corresponding author(s): Ahmet Gorkem Er, Olivier Gevaert

Last updated by author(s): Jan 23, 2024

Reporting Summary

Nature Portfolio wishes to improve the reproducibility of the work that we publish. This form provides structure for consistency and transparency in reporting. For further information on Nature Portfolio policies, see our Editorial Policies and the Editorial Policy Checklist.

Statistics

For all statistical analyses, confirm that the following items are present in the figure legend, table legend, main text, or Methods section.

n/a	Confirmed	
☐	☒	The exact sample size (*n*) for each experimental group/condition, given as a discrete number and unit of measurement
☐	☒	A statement on whether measurements were taken from distinct samples or whether the same sample was measured repeatedly
☐	☒	The statistical test(s) used AND whether they are one- or two-sided<br>*Only common tests should be described solely by name; describe more complex techniques in the Methods section.*
☐	☒	A description of all covariates tested
☐	☒	A description of any assumptions or corrections, such as tests of normality and adjustment for multiple comparisons
☐	☒	A full description of the statistical parameters including central tendency (e.g. means) or other basic estimates (e.g. regression coefficient) AND variation (e.g. standard deviation) or associated estimates of uncertainty (e.g. confidence intervals)
☐	☒	For null hypothesis testing, the test statistic (e.g. *F*, *t*, *r*) with confidence intervals, effect sizes, degrees of freedom and *P* value noted<br>*Give P values as exact values whenever suitable.*
☒	☐	For Bayesian analysis, information on the choice of priors and Markov chain Monte Carlo settings
☐	☒	For hierarchical and complex designs, identification of the appropriate level for tests and full reporting of outcomes
☐	☒	Estimates of effect sizes (e.g. Cohen's *d*, Pearson's *r*), indicating how they were calculated

Our web collection on statistics for biologists contains articles on many of the points above.

Software and code

Policy information about availability of computer code

Data collection	Viral genome sequences were retrieved from the GISAID database. Nextclade CLI (v.2.12.0) was used for sequence alignment and identifying the nucleotide and amino acid mutations and variant clades.
Data analysis	Isolated study strains' phylogenetic tree construction was performed using an optimized substitution model (GTR+F+I) according to the lowest Bayesian Information Criterion (BIC) score obtained by the ModelFinder approach and followed by ultrafast bootstrap analysis (175 iterations) on the IQ-TREE software (v.1.6.12). Consensus tree annotation and visualization were then completed using the ggtree (v.3.8.0) R package. All images were visualized in 3D Slicer (v.5.3.0), and the right and left lungs were segmented with U-net-based pre-trained lungmask R231. 18 first-order, 14 shape, 22 Gray-level co-occurrence matrix (GLCM), 16 Gray-level size zone matrix (GLSZM), and 14 Gray-level dependence matrix (GLDM) features were extracted from original and wavelet-filtered images from each lung using pyradiomics (v.3.0.1). Sparse CCA analysis was conducted using the PMA (v.1.2.1) package. Cooperative learning analyses were performed with the multiview (v.0.8) package .

For manuscripts utilizing custom algorithms or software that are central to the research but not yet described in published literature, software must be made available to editors and reviewers. We strongly encourage code deposition in a community repository (e.g. GitHub). See the Nature Portfolio guidelines for submitting code & software for further information.

Data

Policy information about availability of data

All manuscripts must include a data availability statement. This statement should provide the following information, where applicable:

- Accession codes, unique identifiers, or web links for publicly available datasets
- A description of any restrictions on data availability
- For clinical datasets or third party data, please ensure that the statement adheres to our policy

The data supporting this study's findings are available on request from the corresponding author. The data are not publicly available due to privacy or ethical restrictions. All genome sequences used for training the Word2Vec NLP model and associated metadata in this dataset are published in GISAID's EpiCoV database. To view the contributors of each individual sequence with details such as accession number, virus name, collection date, originating Lab and submitting Lab, and the list of authors, visit 10.55876/gis8.231104eq.

Research involving human participants, their data, or biological material

Policy information about studies with human participants or human data. See also policy information about sex, gender (identity/presentation), and sexual orientation and race, ethnicity and racism.

Reporting on sex and gender	Relevant clinical information was gathered through face-to-face interviews with patients and attending physicians and by reviewing clinical records. Sex was determined based on self-reporting.
Reporting on race, ethnicity, or other socially relevant groupings	Race or ethnicity information was not collected.
Population characteristics	Adult patients (≥18 years of age) hospitalized in the medical wards or intensive care units (ICU) who were positive for SARS-CoV-2 polymerase chain reaction (PCR) test within the last 120 hours and gave informed consent were included in the study.
Recruitment	This study was designed as a prospective cohort study, thus all the patients fill the inclusion criteria were recruited to the study.
Ethics oversight	This prospective cohort study was conducted in a tertiary care academic center in Ankara, Turkey, between December 22, 2020, and May 5, 2021. The cohort consisted of two groups of patients: one group recruited as a part of the project entitled "Viral Genome Analysis in COVID-19 Patients and Genotyping of Genetic Variants Shown in the Literature Related to the Severe Course of the Disease in Humans" and the other group recruited within the scope of the Global Influenza Hospital Surveillance Network Project (GIHSN)-2020-21 in Turkey. Ethical approvals were obtained from the Institutional Ethics Committee with the code numbers GO 2021/02-22, GO 20-102, and GO 22-1211. Good clinical and laboratory practices were followed throughout the study.

Note that full information on the approval of the study protocol must also be provided in the manuscript.

Field-specific reporting

Please select the one below that is the best fit for your research. If you are not sure, read the appropriate sections before making your selection.

☒ Life sciences ☐ Behavioural & social sciences ☐ Ecological, evolutionary & environmental sciences

For a reference copy of the document with all sections, see nature.com/documents/nr-reporting-summary-flat.pdf

Life sciences study design

All studies must disclose on these points even when the disclosure is negative.

Sample size	No sample size calculation was performed.
Data exclusions	Pediatric patients and patients who did not give informed consent were excluded.
Replication	Cooperative learning experiments were replicated to be able to calculate the standard deviation of the results.
Randomization	Patients were not randomized. They were assigned to study groups based on hospitalization places.
Blinding	Blinding was not performed in the study.

Reporting for specific materials, systems and methods

We require information from authors about some types of materials, experimental systems and methods used in many studies. Here, indicate whether each material, system or method listed is relevant to your study. If you are not sure if a list item applies to your research, read the appropriate section before selecting a response.

Materials & experimental systems

n/a	Involved in the study
☒	☐ Antibodies
☒	☐ Eukaryotic cell lines
☒	☐ Palaeontology and archaeology
☒	☐ Animals and other organisms
☐	☒ Clinical data
☒	☐ Dual use research of concern
☒	☐ Plants

Methods

n/a	Involved in the study
☒	☐ ChIP-seq
☒	☐ Flow cytometry
☒	☐ MRI-based neuroimaging

Clinical data

Policy information about clinical studies

All manuscripts should comply with the ICMJE guidelines for publication of clinical research and a completed CONSORT checklist must be included with all submissions.

Clinical trial registration	This study was not a clinical trial. It was designed as an observational study.
Study protocol	Since the study was an observational study, study protocol was only shared with IRB. Online version is not available.
Data collection	This prospective cohort study was conducted in a tertiary care academic center in Ankara, Turkey, between December 22, 2020, and May 5, 2021.
Outcomes	Primary outcome was determined as ICU hospitalization.

Plants

Seed stocks	*Report on the source of all seed stocks or other plant material used. If applicable, state the seed stock centre and catalogue number. If plant specimens were collected from the field, describe the collection location, date and sampling procedures.*
Novel plant genotypes	*Describe the methods by which all novel plant genotypes were produced. This includes those generated by transgenic approaches, gene editing, chemical/radiation-based mutagenesis and hybridization. For transgenic lines, describe the transformation method, the number of independent lines analyzed and the generation upon which experiments were performed. For gene-edited lines, describe the editor used, the endogenous sequence targeted for editing, the targeting guide RNA sequence (if applicable) and how the editor was applied.*
Authentication	*Describe any authentication procedures for each seed stock used or novel genotype generated. Describe any experiments used to assess the effect of a mutation and, where applicable, how potential secondary effects (e.g. second site T-DNA insertions, mosiacism, off-target gene editing) were examined.*
